# Supplementary figures and images for: Demographics and recovery potential of exploited marine teleosts
Source: PLoS One. 2026 Jan 13;21(1):e0340369. doi: 10.1371/journal.pone.0340369 (PMC12799014; doi:10.1371/journal.pone.0340369)

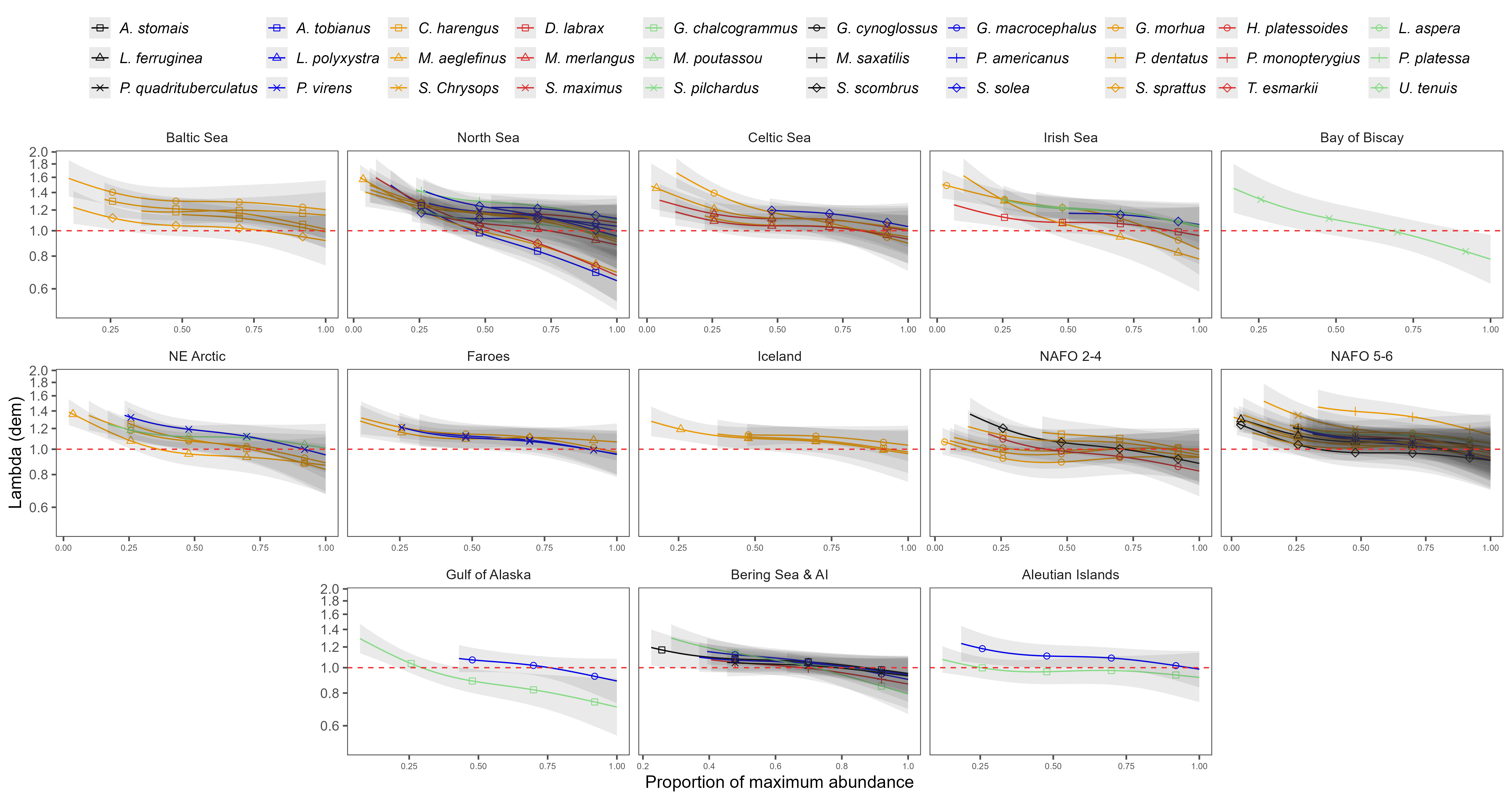

Supplement: S1 Fig — (TIFF) [file pone.0340369.s001.tiff]

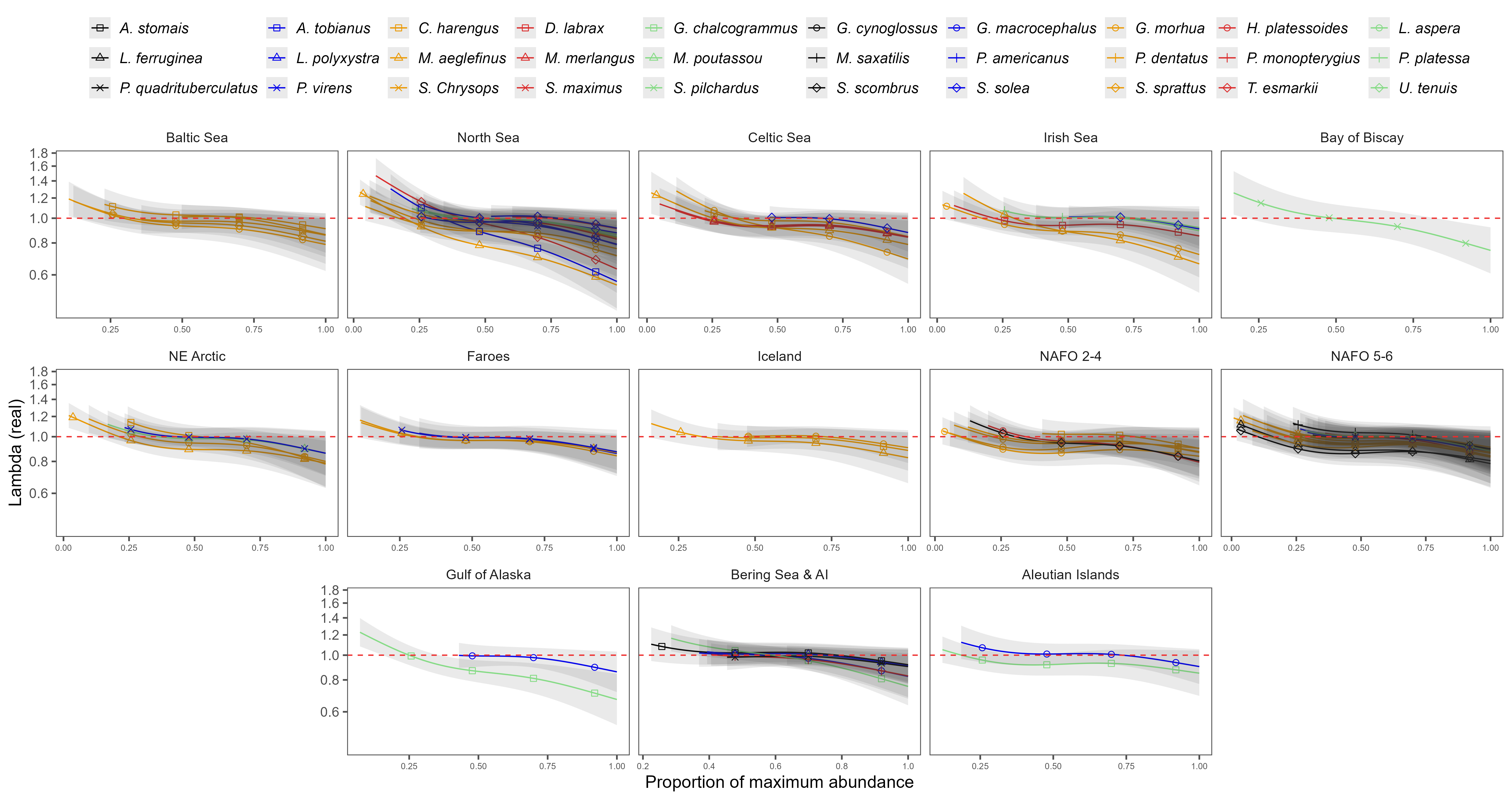

Supplement: S2 Fig — (TIFF) [file pone.0340369.s002.tiff]

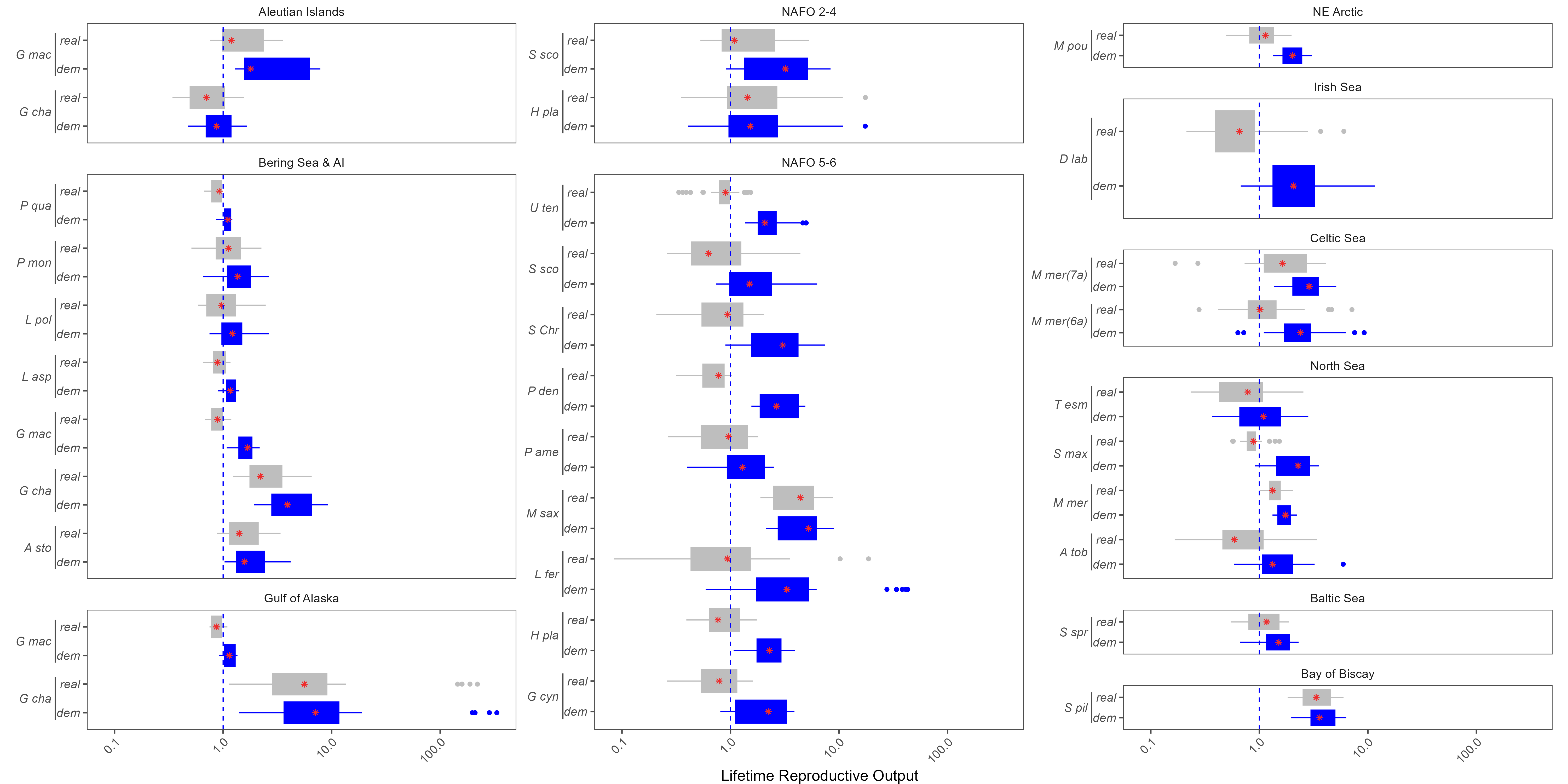

Supplement: S3 Fig — The abbreviated species name is provided on the y-axis. (TIFF) [file pone.0340369.s003.tiff]

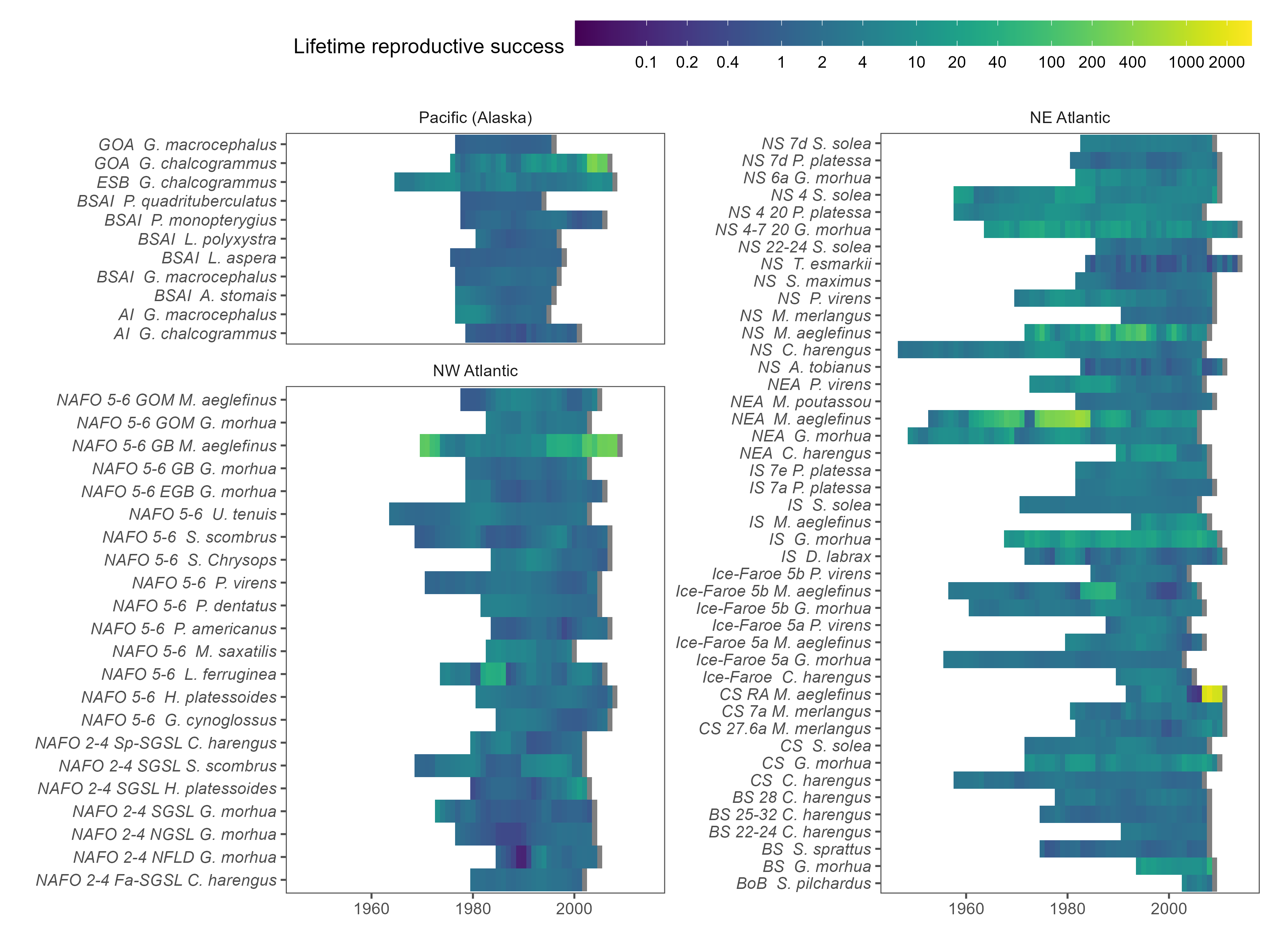

Supplement: S4 Fig — (TIFF) [file pone.0340369.s004.tiff]

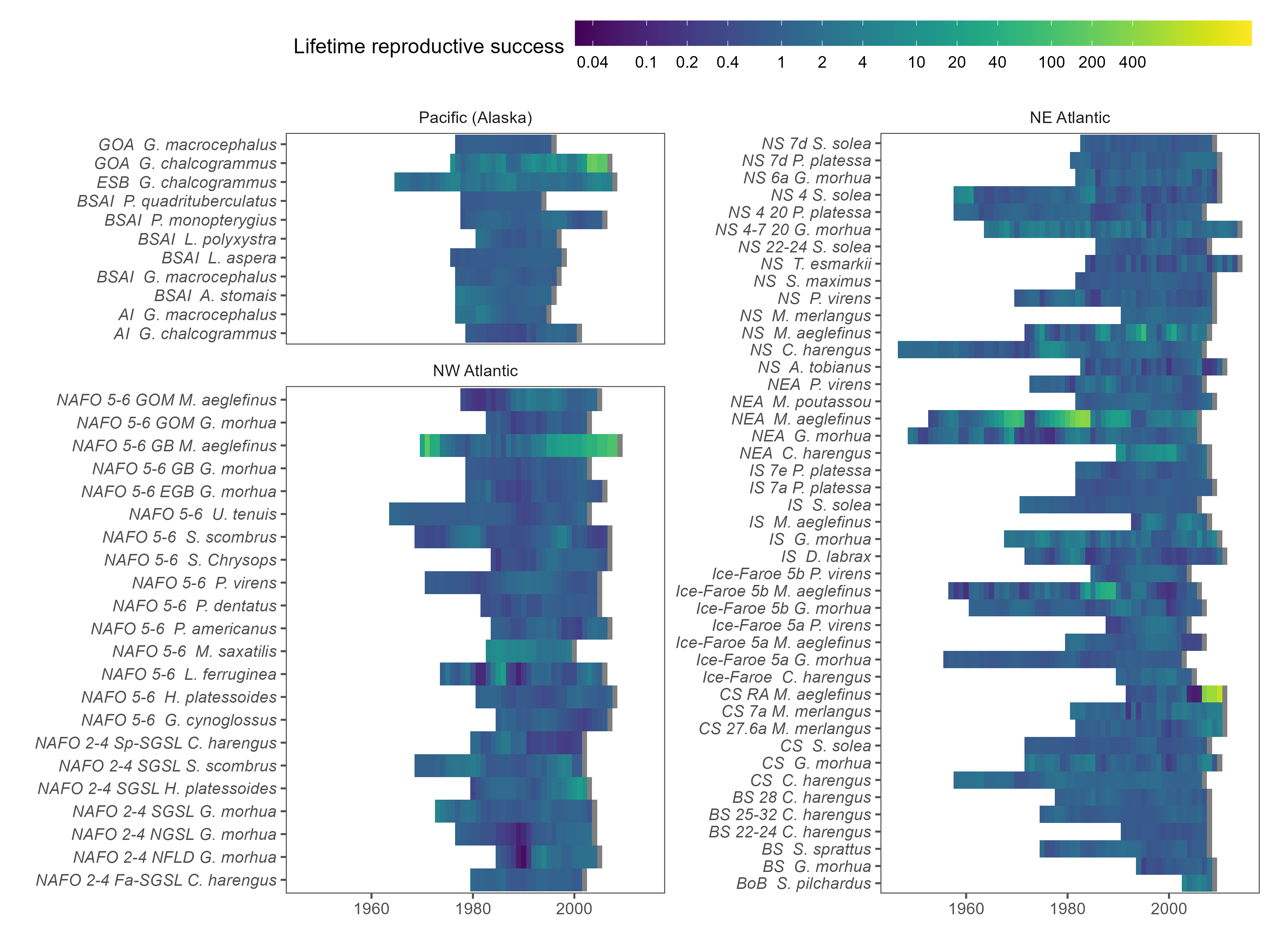

Supplement: S5 Fig — (TIFF) [file pone.0340369.s005.tiff]

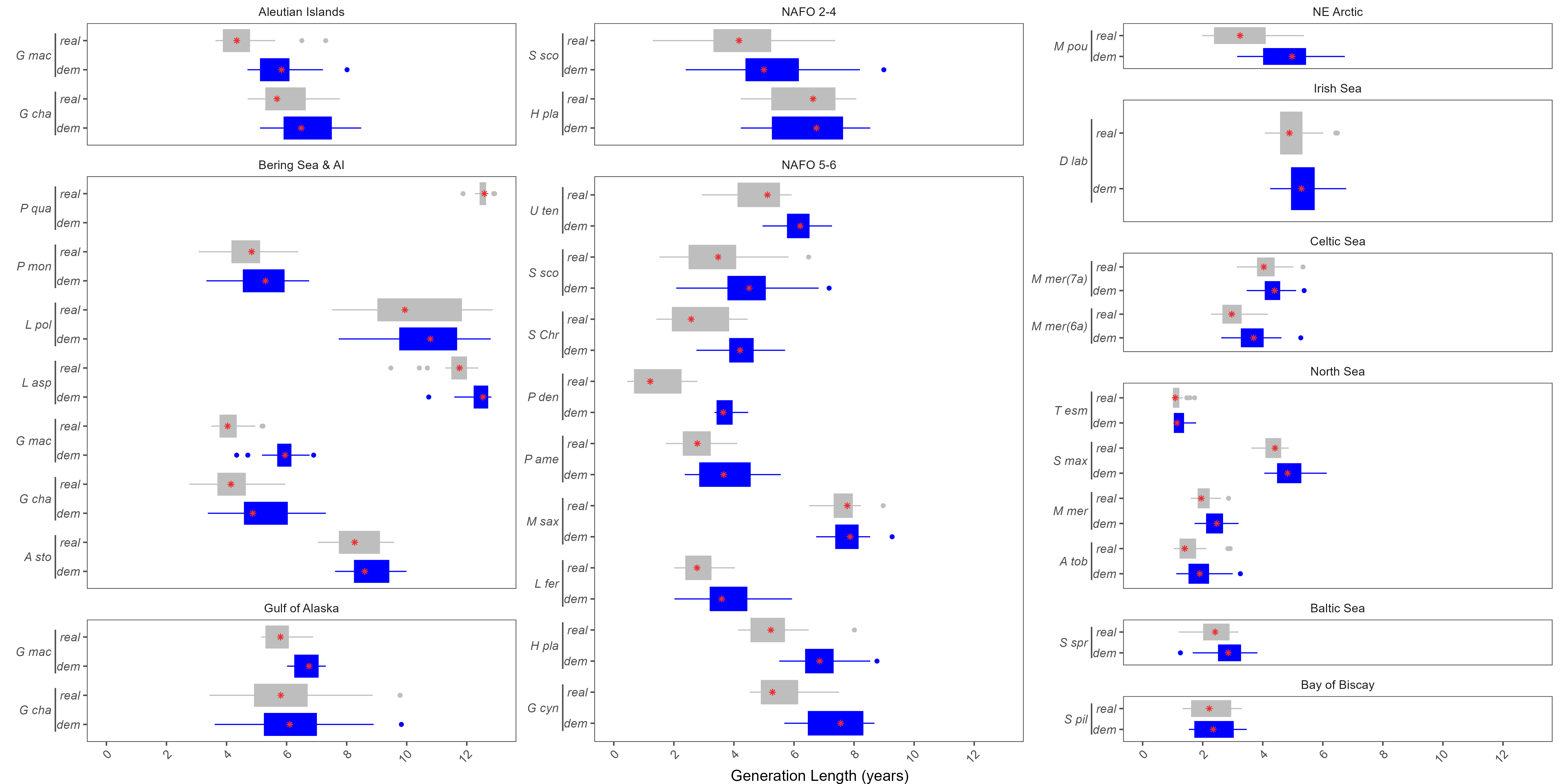

Supplement: S6 Fig — The abbreviated species name is provided on the y-axis. (TIFF) [file pone.0340369.s006.tiff]

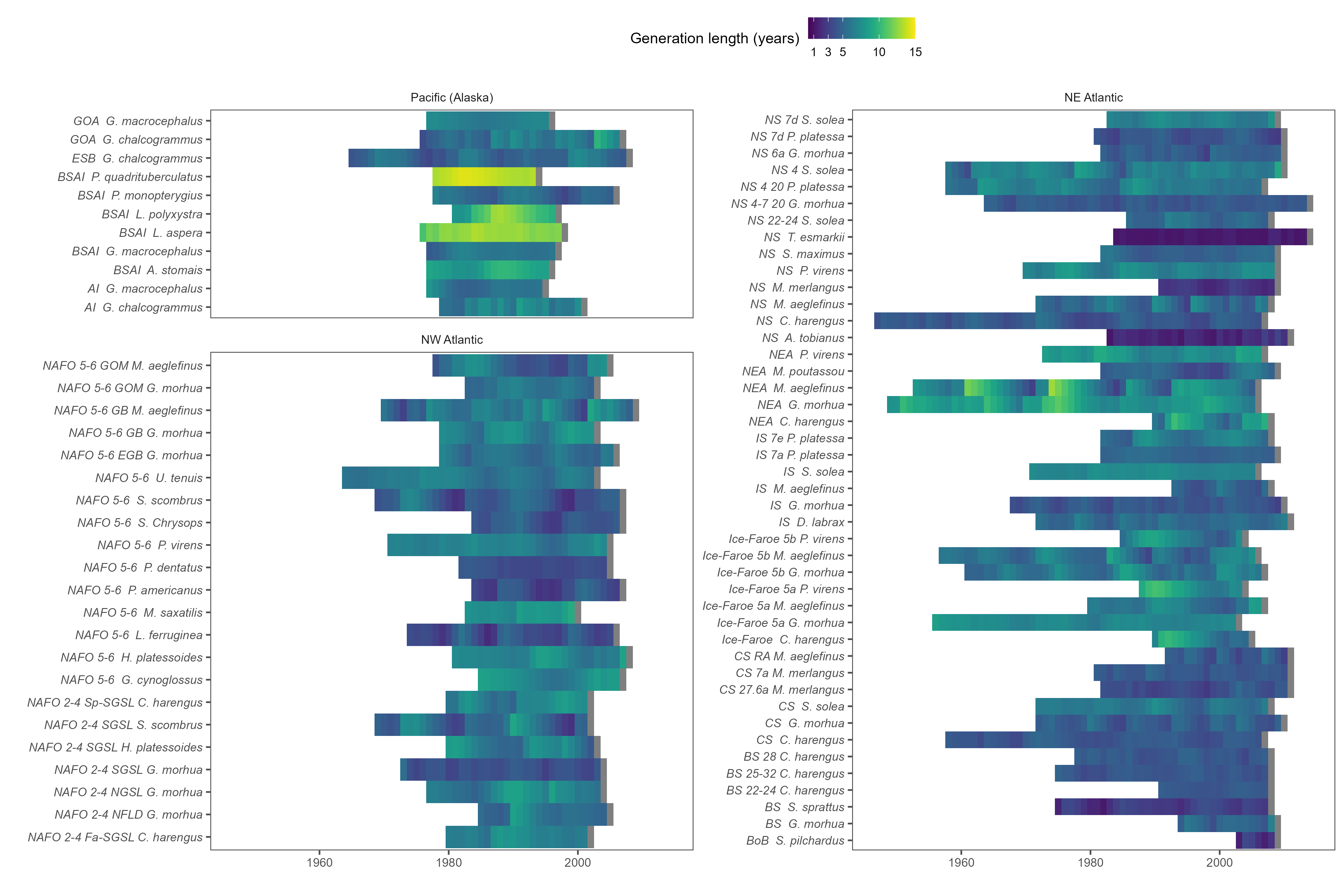

Supplement: S7 Fig — (TIFF) [file pone.0340369.s007.tiff]

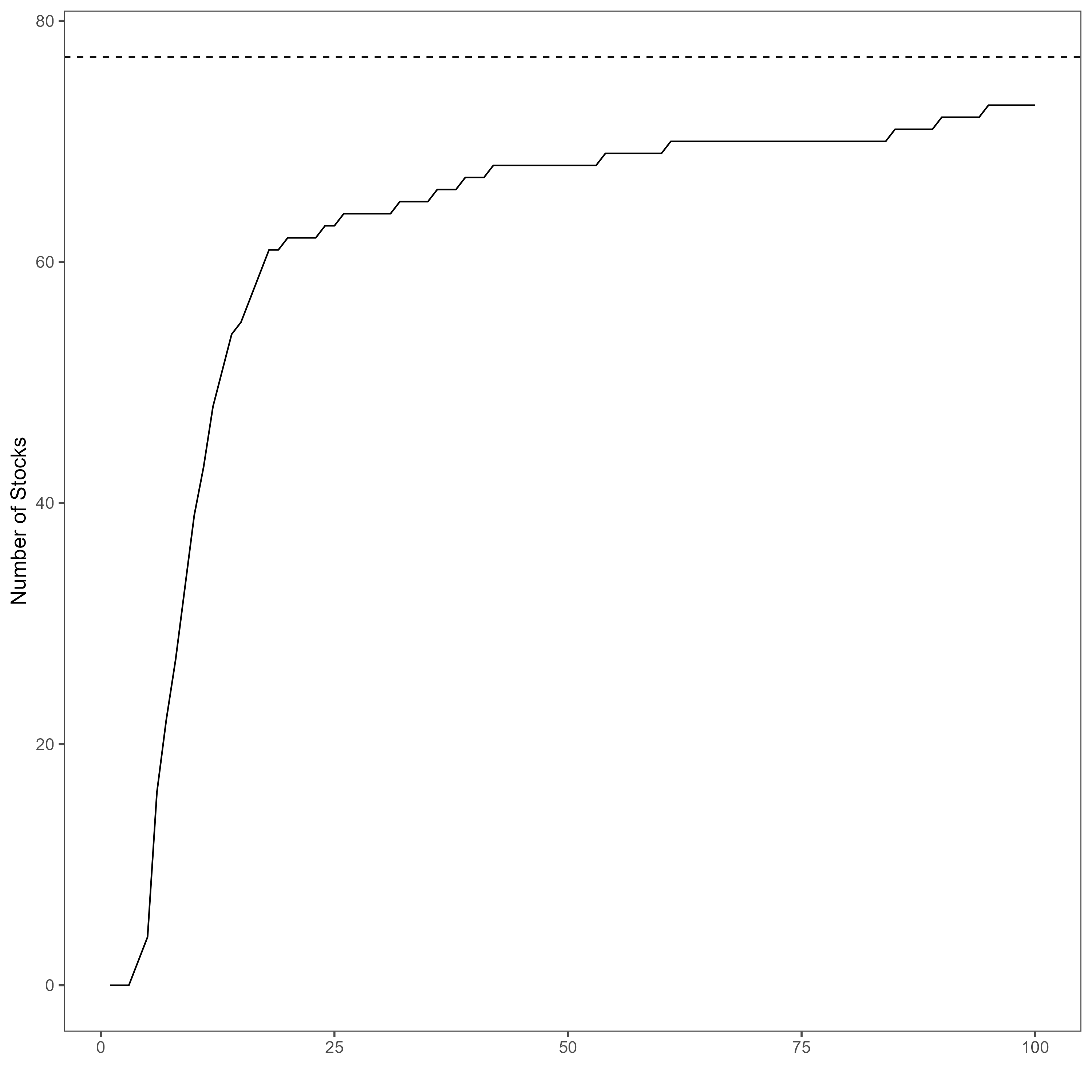

Supplement: S9 Fig — (TIFF) [file pone.0340369.s009.tiff]

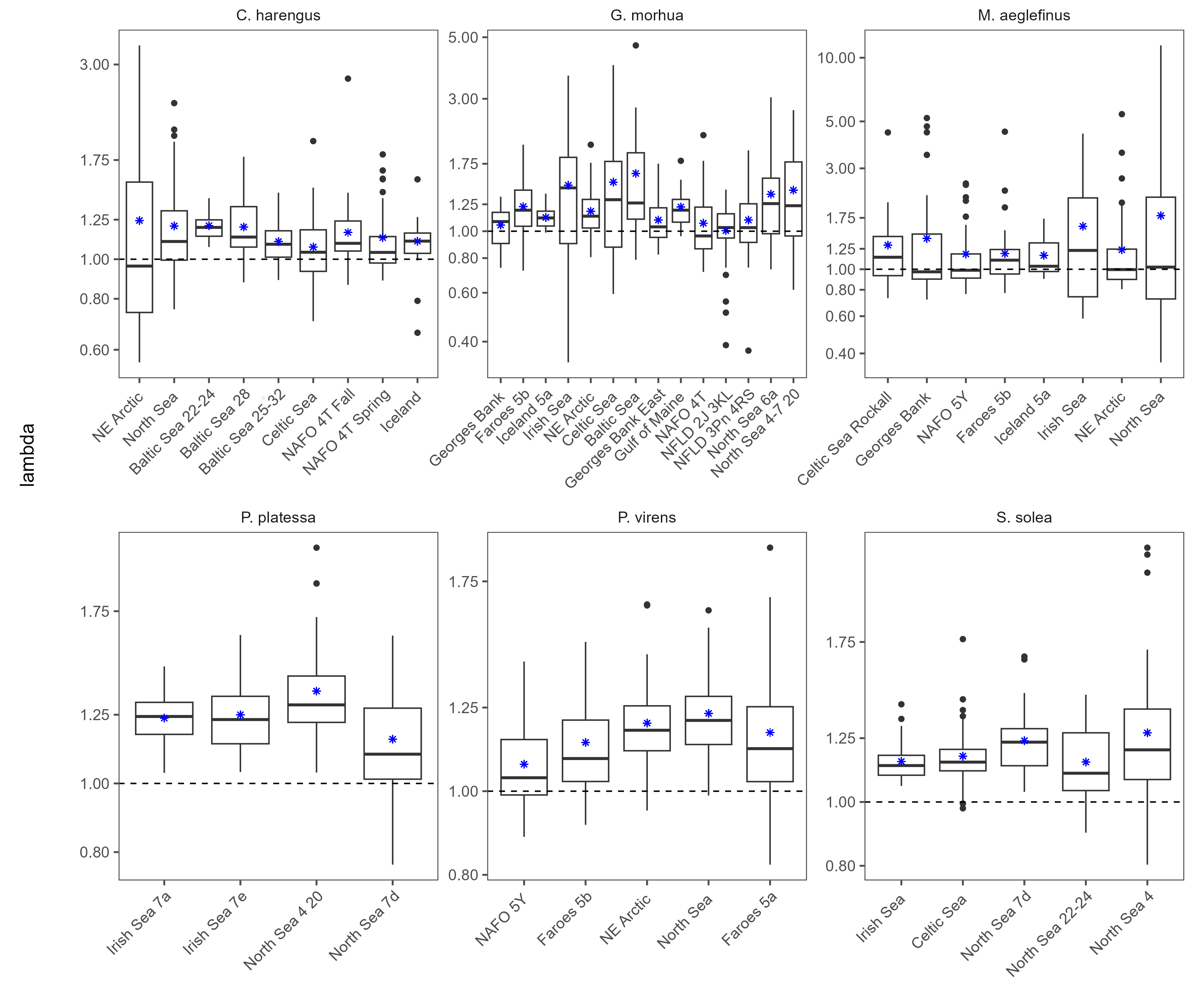

Supplement: S10 Fig — A subest of the results are shown here, with data for each stock of G. morhua, M. aeglefinus, P. virens, C. harengus, P. platessa, and S. solea. The blue stars indicate the mean and the dashed horizontal line represents a λ of one. The y axis is on the log scale. (TIFF) [file pone.0340369.s010.tiff]

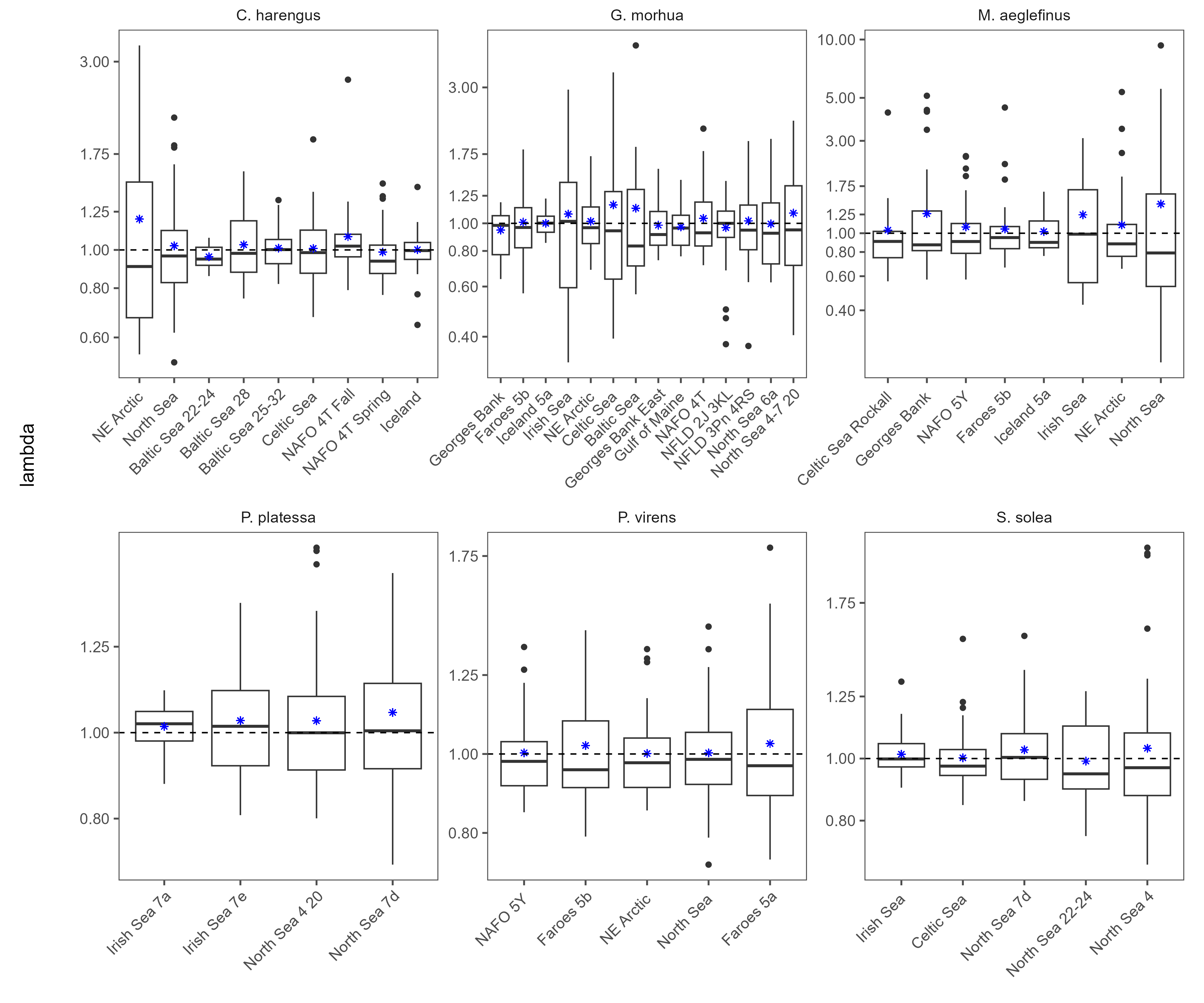

Supplement: S11 Fig — A subest of the results are shown here, with data for each stock of G. morhua, M. aeglefinus, P. virens, C. harengus, P. platessa, and S. solea. The blue stars indicate the mean and the dashed horizontal line represents a λ of one. The y axis is on the log scale. (TIFF) [file pone.0340369.s011.tiff]
