# Supplementary material for: Demographics and recovery potential of exploited marine teleosts
Source: PLoS One. 2026 Jan 13;21(1):e0340369. doi: 10.1371/journal.pone.0340369 (PMC12799014; doi:10.1371/journal.pone.0340369)
Supplement: Attachment — Submitted filename: PLOS_respsonse_to_reviewers.docx [file pone.0340369.s013.docx]

Demographics and recovery potential of globally exploited marine teleosts by Keith et al.

***Reviewer #1:***

In this manuscript, the authors aim to carry out a model-based study to gain insight into the roles of life history traits and density dependence in the population growth, recovery potential, and demographic variability of commercially harvested fish stocks in the North Atlantic and North Pacific. Specifically, this study evaluates population and demographic responses to fishing pressure for 77 marine fish stocks in the regions by reconstructing life tables using Leslie matrices with annual demographic parameters (fecundity and survivorship) estimated from age-structured stock assessment model outputs. The authors conclude that most of the stocks in the study (nearly 90%) have a good recovery potential, but both population growth and recover potential can be highly variable among stocks, indicating weak evidence of life history characteristics’ roles in population and demographic variability.

Overall, this is a very well-written manuscript with clearly stated objectives that are worthy of investigation. I believe that the authors present interesting, solid work that provides much needed information in evaluating the recovery potential of harvested fish stocks exposed to varying levels of fishing pressure. While the roles of demographic variability and density dependence in the population dynamics of exploited fish species have been explored in past research, by applying the life table approach to a large number of stocks in several regions this study provides further evidence that the commonly held view of life history theory may need to be reconsidered when applied to harvested species. I just have a few minor comments below.

*We thank the reviewer for their thoughtful comments and have addressed their comments below and revised the manuscript as outlined in our response.*

*Specific comments:*

l.19: ‘stocks’ -> ‘stocks with’

*Revised, thanks!*

l.98: Has this repository become publicly available?

*Yes, an oversight on my part resulted in the* [*repository*](https://github.com/JulieCharbonneau/Age-structured-marine-fish-database) *not being updated with the latest data, this is now rectified.*

l.126-l.129: I would suggest the authors add the results of this test to the Supplementary material.

*We have included two figures in the supplement showing how similar the results are (Figures A10 and A11).*

l.231: Was density dependence tested on lambda in the same year? I would imagine there is a time lag of a year (or more) at least in some stocks.

*It was tested using the difference in the abundance (i.e., lambda in 2025 utilizes the difference in abundance between 2024 and 2025). The challenge with testing this across years is that lambda is an integrated metric accounting for recruitment, growth, and total mortality (both natural and fishing mortality), thus we felt that measuring lambda in the year in which the dynamics were observed was most appropriate. We agree that time lags would be a factor for many of these stocks (e.g.,* [*Charbonneau et al. 2022*](https://cdnsciencepub.com/doi/full/10.1139/cjfas-2021-0338)*) and we are currently undertaking research that attempts to better understand how temporal correlation, along with correlations between stocks, may be impacting these dynamics.*

l.251: Does the interaction here mean ‘Stock’ and ‘Prop’?

*Page 6 Line 5: Yes, we have revised the text to clarify, thanks for catching that.*

l.347: ‘it’s’ -> ‘its’
*Revised*

l.406: ‘stock’ -> ‘stocks’.
*Revised*

l.518: ‘comnpare’ -> ‘compare’

*Revised*

***Reviewer #2:***

The authors apply the Lotka–Euler equation to life-history schedules derived from stock assessment outputs to estimate demographic growth capacity for 77 stocks in the North Atlantic and Northeast Pacific, both including and excluding the effects of fishing mortality. They also test whether the populations show signs of density dependent growth rate and they also estimated various demographic parameters, like lifetime reproductive success, generation and doubling time. Based on these estimates, they comment on the innate capacity of these stocks to recover as well as on the drivers of variation in population productivity under different levels of exploitation and density conditions. Overall, the manuscript addresses an important question: how well demographic life-table metrics deriving from standard stock assessment outputs reflect the potential for growth and recovery in exploited marine fish populations. The idea is timely, the analyses are methodologically sound, and the authors are careful in presenting caveats associated with their approach.
In any case, the weaknesses of this analysis are explicitly pointed out by the authors themselves. Probably the most striking outcome is the almost complete lack of intra- and interspecific patterns consistent with life-history theory expectations. For example, slow growing, high trophic level species like cod or haddock showed comparable or even higher growth capacity and recovery potential than fast growing, lower trophic level species such as herring or sardine. This has led the authors to acknowledge that probably “equilibrium theory and general life-history characteristics […] are poor predictors of population growth rates and recovery potential.”
However, probably the main reason for these inconsistencies does not lie in the authors’ analytical approach, but in the nature of the input data. Stock assessments are designed primarily to provide management-relevant estimates of biomass, fishing mortality, and abundance trends. While model diagnostics focus on statistical fit, assessment models often rely on biological assumptions that are not always inspected or validated, potentially resulting in biologically unrealistic representations of population dynamics. Moreover, outputs such as abundance-at-age, maturity, natural mortality, and survival are influenced by model structure, data limitations, retrospective adjustments, and exploitation history. These limitations are acknowledged by the authors in several parts of the manuscript, particularly in the final paragraph of the Discussion.
Nonetheless, these limitations do not diminish the value of the manuscript. The underlying idea is solid and interesting, the analyses are robust, and the text is well written and clearly justified. Importantly, the authors show that under current management regimes stocks still rely on recruitment bonanzas for retaining production capacity, a critical message for designing sustainable management strategies. Their density-dependence analysis is also interesting and in line with ecological and demographic theory.
Thus, I recommend publication of this paper after revision.

*Thank you for the thorough review and insightful comments, especially regarding the temporal trends in lambda. We did look at this previously but moved on from the analysis as we did not notice any obvious trends. However, we had been focusing on a species by species level, and based on your suggestion we revisited this using a more global perspective. We found there does seem to be an interesting decline in lambda in the 2000s and 2010s that we did not notice previously. We have added a paragraph in the results and discussion outlining these results. Thank you for the idea; it adds an interesting nuance to the paper.*

Specific Comments
Title
The authors analyze 77 stocks from the North Atlantic and Northeast Pacific Oceans. Given this regional coverage, the phrase “globally exploited marine teleosts” overstates the spatial extent and representativeness of the dataset. I recommend rephrasing the title to better reflect the actual geographical scope of the analysis.

*Fair point, we have removed the word global from the title.*

Lines 83 – 102:
The dataset used by the authors (Charbonneau and Keith 2022), available through the GitHub repository cited in the corresponding reference, contains information on abundance, weight-at-age, fishing mortality, and Fmsy (for some stocks) for 101 stocks. This dataset does not seem to include information on maturity, natural mortality and fishery removals, so it would be better if the authors explained the source for this data too. In addition, since that dataset includes more stocks than the 77 used here, please explain the criteria or data filtering steps that led to your final selection.

*Yes, an oversight on my part resulted in the* [*repository*](https://github.com/JulieCharbonneau/Age-structured-marine-fish-database) *not being updated with the latest data, this is now rectified. The 77 stocks used in this analysis contained sufficient age-structured data to undertake the analysis; the other stocks in the complete database were missing information about at least one of maturity-at-age, fishery removals-at-age, weight-at-age, or abundance-at-age.*

Line 112:
Since you apply the discrete time version of the Euler-Lotka equation, I don’t think that you need dx in your equation.

*Page 3, Line 31: We surely do not, thank you for catching that, it has been revised to Δx (which is always 1 for this analysis).*

Lines 160 – 187:
The authors refer to fishing mortality, natural mortality and total mortality, but the values used in the Euler–Lotka equations are actually annual proportional survival or removal rates (bounded between 0 and 1), rather than instantaneous mortality rates (F, M, Z) in the standard continuous-time fisheries sense/stock assessment routine.
So, it would be important for the authors to explicitly state that mortality inputs were transformed into proportional form before being used in the life-table calculations to help avoid possible confusion, especially for readers accustomed to interpreting F, M, and Z as instantaneous rates in stock assessment models.

*Page 5 Line 11: Excellent point, we have revised the text to clarify that both fishing and natural mortality were proportions.*

Lines 360 – 361:
A verb is missing from the sentence “The changes in the rate of…”

*Revised, thanks!*

Lines 435 – 436:
The authors state that “when the effects of fishing were excluded, these results indicated these stocks retained an innate capacity for increase of approximately 8.7%”. This value is derived from the median population growth rate when excluding the effects of fishing for the 77 stocks (lines 321-322). However, λdem, as correctly pointed out by the authors, is not independent from fishing pressure and by no means represent the maximum intrinsic rate of growth for populations.
By relying on the median λdem over the full time series, the authors implicitly assume that fishing pressure, status and management of stocks have been relatively stable throughout the nearly 40 years analyzed. However, no information is provided on whether there are temporal trends for λ stock values. If there is an increasing or decreasing trend for λ (which seems plausible based on changing exploitation levels or even environmental conditions), then the median across all years may not reflect the current innate capacity for population change.
In that case, probably the current (or recent) capacity of the populations cannot support an 8.7% increase. If such temporal trends actually exist, then a metric based on more recent years (e.g., the mean or median of the last three to five years) may offer a more accurate estimate of present-day demographic potential. I suggest authors to examine and report whether temporal trends exist and possibly revise their assumptions accordingly.

*Thank you for this, we agree that exploring the temporal variability in the lambda’s is a very good idea. It is somewhat challenging to compare across the early time periods as there are few stocks in the analysis, but we feel that reasonable comparisons can be made from the 1980s to the 2010s as ≈90% of the stocks have data back into the 1980s. The results are also interesting, the abundance (proportion of maximum) is lower in the 2000s and 2010s, however, the lambda values are lower than in previous decades. The significance of the relationship using formal statistical tests is highly dependent on the starting year, so we’re a bit hesitant to overstate the results here, but this is hinting at a decline in productivity over the last couple of decades.*

*Page 10 Lines 12-22 & Page 21 Lines 3-21: We have added a paragraph in the results and discussion related to the temporal trends, along with a table summarizing the median/mean and variation of lambda by decade. This is a thread we are interested in exploring in our research moving forward, so thank you very much for highlighting this.*

Line 518:
Correct the typo “comnpare”

*Revised, thanks!*

Tables 1 and 2:
I assume that the authors chose to estimate and present λ at 40% of maximum abundance because this is the threshold where the density independent part of the model begins. However, this is not stated explicitly anywhere in the manuscript, nor in the tables captions. I suggest to add a note (within the text or/and in table caption) explaining the choice of 40% estimations.

*Page 7, Lines 13-16: Thanks for catching that. We’ve added a sentence into the methods indicating that we used 40% due to both theoretical expectations and observed population dynamics.*

Figures:
Please italicize the species names.

*Done!*
